# GenePANDA—a novel network-based gene prioritizing tool for complex diseases

**DOI:** 10.1038/srep43258

**Published:** 2017-03-02

**Authors:** Tianshu Yin, Shu Chen, Xiaohui Wu, Weidong Tian

**Affiliations:** 1 State Key Laboratory of Genetic Engineering and Collaborative Innovation Center for Genetics and Development, School of Life Sciences, Fudan University, Shanghai 200436, P. R. China; 2 Department of Biostatistics and Computational Biology, School of Life Sciences, Fudan University, Shanghai 200436, P. R. China; 3 National Center for International Research of Development and Disease, Institute of Developmental Biology and Molecular Medicine, Fudan University, Shanghai 200433, P. R. China

## Abstract

Here we describe GenePANDA, a novel network-based tool for prioritizing candidate disease genes. GenePANDA assesses whether a gene is likely a candidate disease gene based on its relative distance to known disease genes in a functional association network. A unique feature of GenePANDA is the introduction of adjusted network distance derived by normalizing the raw network distance between two genes with their respective mean raw network distance to all other genes in the network. The use of adjusted network distance significantly improves GenePANDA’s performance on prioritizing complex disease genes. GenePANDA achieves superior performance over five previously published algorithms for prioritizing disease genes. Finally, GenePANDA can assist in prioritizing functionally important SNPs identified by GWAS.

One major challenge in human genetics is to identify the genetic causes underlying complex diseases. The discovery of disease genes often starts with a cytogenetic study, a linkage analysis, or a genome-wide association study (GWAS)^1^,^2^,^3^. Without prior knowledge about the disease, however, the study size must be large enough to demonstrate the significance of findings, after accounting for multiple hypothesis testing. Knowledge about which genes are most likely to be involved in the disease, *a priori*, can significantly reduce the number of hypotheses, which in turn reduces the study size at a given power. This is the so-called “candidate-gene approach”^4^. For example, given that defects in DNA damage response and DNA repair have strong association with skin cancer^5^,^6^, instead of performing an exhaustive whole genome search, we could simply focus on genes involved in those pathways. On the other hand, many genetic variants found in GWAS were suspected to be false discoveries due to experimental design or analytical issues^7^,^8^. Such genetic variants could be readily filtered out if we had prior knowledge about their association with the disease. In the past, candidate disease genes from a specific pathway were determined manually by geneticists and biologists based on their knowledge and expertise. As an example, nine genes in a manually compiled pathway centered on interleukin (IL)-12 and IL-23^9^,^10^ were identified as susceptibility genes for Crohn’s disease in various replication and association studies^11^,^12^,^13^,^14^,^15^,^16^. However, current knowledge about a specific pathway is often not complete, which has limited the application of the candidate gene approach.

Given the rich trove of functional genomics data in public domains, various computational methods have been developed to predict or evaluate whether a gene is likely a candidate disease gene, which is often called disease gene prioritization^17^,^18^. Based on their strategies for prioritizing candidate disease genes, current methods can be generally classified into three categories—text mining, similarity profiling, and network analysis-based methods. Text mining-based methods rely on the use of biomedical literature sources to identify co-occurrence of both already known disease genes and promising candidate genes using statistical methods. For example, aBandApart^19^ and Gene Prospector^20^ both mine MEDLINE data to uncover candidate disease associated genes. However, for most genes they may not have been reported in the same literatures with known disease genes; consequently, their association with diseases could not be uncovered through text mining. Similarity profiling-based methods, such as Endeavour^21^ and ToppGene^22^, typically employ machine-learning approaches to integrate multiple sources of genomics evidence to identify candidate disease genes that have similar patterns to the profile of a set of genes, keywords, functional annotations, gene expression already known to be associated with a given disease. Network analysis-based methods are also based on the use of multiple sources of genomics evidence, except that these data are usually presented in the form of functional association network. These methods typically predict candidate disease genes by measuring their network characteristics (correlation, connectivity, distance, etc.) to known disease genes from different perspectives, such as Pinta^23^, Maxlink^24^ and Genefriends^25^.

In this study, we presented a novel network analysis-based method named GenePANDA (Gene Prioritizing Approach using Network Distance Analysis) for prioritizing candidate disease genes. The network used by GenePANDA is the STRING network, a probabilistic functional association network constructed by various sources of experimental and predicted gene associations (Franceschini, *et al*.^26^). A unique feature of GenePANDA is the introduction of adjusted network distance that is derived by considering not only the direct network distance between two genes, but also their respective mean network distances to all other genes in the network. Based on the adjusted network distances, GenePANDA scores a candidate disease gene by measuring its distance to disease genes relative to random genes in the network. The use of adjust network distance proved to significantly improve the performance of GenePANDA when it was applied to 196 complex diseases. GenePANDA was also compared with three network analysis-based methods—Genefriends, Maxlink and Pinta, and two similarity profiling-based methods—Endeavour and Candid using two benchmarks, and showed superior performance. Finally, GenePANDA was applied for prioritizing non-synonymous single nucleotide polymorphisms (SNPs) identified in a number of genome-wide association studies. A free web-based implementation of GenePANDA is available at http://genepanda.tianlab.cn, where researchers could input a list of interesting genes associated with a given disease or phenotype, and then quickly receive a ranked list of candidate genes.

## Materials and Methods

### Data Sources

#### The Gene Network

The STRING network^26^ (http://string-db.org/, version 9.1) was used as the reference network for GenePANDA. It consists of 19,038 protein-coding genes and over 4.8 million weighted edges that represent either known or predicted interactions between a pair of proteins. The predicted interactions are derived from four sources: genomic context, high-throughput experiment, co-expression and previous knowledge.

#### Disease-related Gene Sets

Genetic Association Database (GAD, http://geneticassociationdb.nih.gov/, version in Jan, 2013) is a widely recognized database that includes curated summary data from previous work and primarily focused on archiving information on common complex human disease^27^. We select the GAD database as the resource of known disease genes for complex diseases. The latest version of GAD includes the annotation of associated genes for 200 complex diseases. We assume that all gene-disease associations annotated by the GAD database are true associations, and select 196 diseases that have at least 2 associated disease genes for prediction.

#### Disease-related SNPs

We obtained the SNP data for the following diseases from the respective websites: Crohn’s disease (International IBD Genetics Consortium^28^ (http://www.ibdgenetics.org/downloads.html)), obesity (GIANT consortium^29^, (https://www.broadinstitute.org/collaboration/giant/images/5/5e/GIANT_Yang2012Nature_publicrelease_HapMapCeuFreq_BMI.txt.gz), rheumatoid arthritis^30^ (http://www.broadinstitute.org/ftp/pub/rheumatoid_arthritis/Stahl_etal_2010NG/). Here, in each dataset we selected non-synonymous SNPs with p-value less than 5*10^−8 (genome-level significant threshold), and then mapped SNPs to their corresponding genes for subsequent studies via Ensembl Variant Effect Predictor^31^.

### Algorithm design of GenePANDA

The algorithm of GenePANDA consists of three steps: (i) network distance computation and adjustment, (ii) disease-specific gene weighting, and (iii) score conversion.

#### Network distance computation and adjustment

The STRING network is a weighted network, with each edge assigned a score *S* ranging from 0 to 1000, representing the confidence of functional interaction between the two genes, with higher score indicating higher confidence about the interaction (e.g., according to the STRING website, score > 900, score > = 700, score > = 400, score > = 150, and score < 150 represent highest confidence, high confidence or better, medium confidence or better, low confidence or better and below low confidence about the interaction, respectively). The raw network distance between two genes with a link in the network is defined as *D *=* 1000/S*, such that a smaller D (shorter distance) would correspond to a higher confidence of functional interaction. For those pairs of genes that are not directly linked in the network, their network raw distance is defined as the shortest path between the two genes in the network based on the Djikstra’s algorithm^32^.

A key step in GenePANDA is the computation of the adjusted network distance. Given the raw network distance between gene *a* and gene *b, D*_*ab*_, we then compute their adjusted network distance 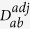 as: 
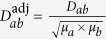
, where *μ*_*a*_ and *μ*_*b*_ are the mean raw network distances for *a* and *b*, respectively. The mean raw network distance for *a* is defined as 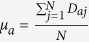, where N is the total number of genes (19,038) in the network, and *D*_*aj*_ is the raw network distance between gene *a* and gene *j (D*_*aa*_ = 0).

#### Disease-specific gene weighting

Given a list of known disease genes, we reason that a candidate disease gene should have stronger functional interaction with known disease genes than with random genes in the network. Thus, we introduce a disease-specific gene weight, *w*_*i*_, defined as the follows:


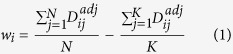


where *N* is the total number of genes in the network, *K* is the total number of disease genes, and 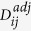 is the adjusted network distance between gene *i* and gene *j*. In this formula, the first and the second component correspond to the mean adjusted network distance of gene *i* to all genes in the network and to all known disease genes, respectively. It can be easily seen that a larger *w*_*i*_ would suggest that the gene under investigation has relatively shorter distance (stronger functional interaction) to disease genes than to random genes, is therefore more likely to be a candidate disease gene.

#### Score conversion

For a given disease, the disease-specific gene weights can be compared with each other, with higher weight indicating higher probability to be a candidate disease gene. However, they cannot be directly compared across diseases. To make them comparable across diseases, we apply a score conversion procedure to convert the weights into probabilities. For a given disease, we first sort all genes by ranking *w*_*i*_ in descending order. Then, at each *w*_*i*_ we calculate the corresponding precision defined as *Precision* = *TP*/*P*, where *TP* and *P* are the total number of disease genes and the total number of all genes with a weight above *w*_*i*_, respectively. The precision score is the probability that a gene with a weight above *w*_*i*_ is likely to be a disease gene, and can therefore be compared across different diseases.

### Benchmark the Performance of GenePANDA

Currently, there are only few methods that prioritize disease genes at genome-scale^33^. Thus, we select only five of them to compare with GenePANDA, which are Genefriends^25^, Maxlink^24^, Pinta^23^, Candid^34^, and Endeavour^21^. Genefriends and Maxlink, Pinta are network analysis-based methods, while Candid and Endeavour belong to similarity profiling-based methods. Here, we briefly describe the algorithm of each of these five methods. For details about each method, refer to the respective publication. Genefriends employs a guilt-by-association approach to prioritize candidate genes based on their co-expression level with known disease genes in a co-expression network. Maxlink ranks candidate cancer gene based on their network connectivity to known cancer genes in FunCoup, a probabilistic functional association network. Candid mines several heterogeneous data sources such as literature, protein domains, conservation and expression, and assigns criterion-specific scores to each gene, which are then normalized and summed to form the gene’s final score. Endeavour prioritizes candidate genes based on their similarity to known disease genes by integrating information from multiple biomedical data sources. Pinta prioritizes candidate genes in a genome-wide protein-protein interaction network by inspecting the degree of differential expression in their neighborhood.

We prepare two benchmark datasets to compare GenePANDA with the above-described five methods. Benchmark dataset 1 consists of two independent gene lists. One is aging related gene list used for benchmarking Genefriends, and includes 229 over-expressed genes found in mammal meta-analysis of age-related gene expression profiles^35^. The other is a list of cancer-related genes from Cancer Gene Census compiled by Maxlink^36^.

Though Pinta is also a network analysis-based method, it requires the input of gene expression data, and is therefore not compared here. To evaluate the performance of different methods, in this benchmark we use the true-positive rate measure when setting different threshold, this is to estimate how efficient the tools are if only the top candidate genes would be assayed in the real situation. Recently, Bornigen *et al*. prepared a collection of 42 lists of novel disease-gene associations, and used these associations as unbiased validation for evaluating the performance of different gene prioritization methods^33^. They also provided a list of disease genes for predicting each of the 42 disease-gene associations, and evaluated the performance of Endeavour-GW, Candid and Pinta-GW (GW is short for genome-wide). Here, we use the datasets prepared by Bornigen *et al*. as benchmark 2, and run GenePANDA to predict the 42 disease-gene associations. We follow Bornigen *et al*. to use the rank ratio (ranking position) of the 42 novel disease-gene associations to measure the performance of GenePANDA, and compared it with that Endeavour-GW, Candid and Pinta-GW of reported by Bornigen *et al*.

## Results

### A brief overview of the algorithm design of GenePANDA

**Figure 1:**
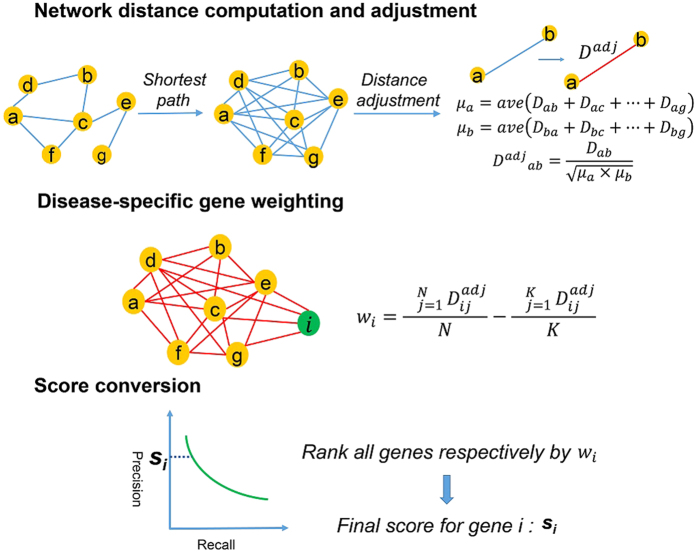
The workflow of GenePANDA. It basically comprises of three steps: network distance computation and adjustment, disease-specific gene weighting, and score conversion. For details about each step, refer to the Method section.

GenePANDA (**Gene P**rioritization using **A N**etwork-**D**istance based **A**pproach) consists of three major steps: calculation of adjusted network distances, calculation of disease-specific gene weights, and conversion of gene weights into probabilities (Fig. 1). For details regarding to the algorithm design of GenePANDA, refer to the Method section. The calculation of adjusted network distance is a key step in GenePANDA, which is done by considering not only the raw network distance between two genes in the network, but also their respective mean raw network distance to all other genes in the network. The rational is that the significance of a functional interaction should be dependent on not only the interaction itself, but also the centrality properties of the two interacting genes. The calculation of disease-specific gene weights is based on the hypothesis that a candidate disease gene should have stronger functional interaction (smaller network distance) to known disease genes than to random genes in the network. Finally, the purpose for score conversion is to make the prediction scores comparable across diseases.

### The use of adjusted network distance significantly improves GenePANDA’s performance on prioritizing disease genes

**Figure 2:**
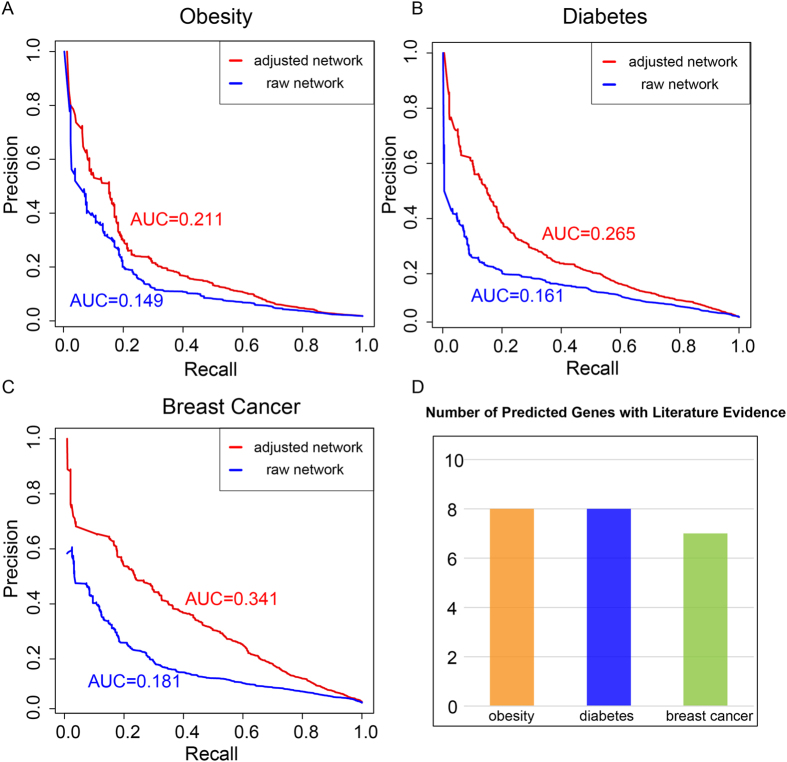
Prediction performance of GenePANDA on three diseases. Leave-one-out cross validation is conducted. The Precision-Recall curve of GenePANDA on obesity, diabetes, and breast cancer using adjusted distance vs raw distance is shown in (**A**), (**B**), and (**C**), respectively. Among the top 10 predictions for these three diseases, the numbers of predictions with literature support are shown in (**D**).

We obtain all disease gene annotations from Genetic Association Database (GAD)^37^. Then, we first select three complex diseases: obesity, diabetes and breast cancer that have 335, 825 and 786 known disease genes, respectively. We conduct leave-one-out cross validation to prioritize disease genes for each of these three diseases by using the adjusted network distance, and then compare the performance with that obtained by using the raw network distance. It can be clearly seen that the use of adjusted network distance significantly improves the performance of GenePANDA for all three diseases (Fig. 2A–C): the AUC of the precision-recall curve for obesity, diabetes, and breast cancer based on the adjusted network distance are 0.211, 0.265, and 0.341, respectively, all significantly higher than that based on the raw network distance (0.149, 0.161, and 0.181, respectively).

When evaluating the performance of GenePANDA in these three diseases, we assume those genes that are not currently annotated as the disease genes as false predictions. In reality, the top “false” predictions would be considered as candidate disease genes because they have strong functional interactions with known disease genes. Here, we rank all “false” predictions based on their prediction scores, and select the top 10 genes for literature validation using papers published after year of 2014. Out of 10 predicted genes for obesity, diabetes and breast cancer, 8, 8 and 7 are validated by literature reports published recently (Fig. 2D, detailed literature evidence can be in Supplementary Table S1), proving the usefulness of GenePANDA for disease gene prioritization. Below we provide an example for each of the predictions for the three diseases. *GCG* is the top predicted gene for obesity. Glucagon (GCG), is a pancreatic hormone that counteracts the glucose-lowering action of insulin by stimulating glycogenolysis and gluconeogenesis^38^. In 2015 it was reported that among obese patients who underwent surgery of Roux-en-Y gastric bypass (RYGB) for weight loss, GCG was expressed at significantly higher level and was suggested to play a role in the improved glycaemic and metabolic status of obese patients^39^. It is therefore likely that abnormal expression of *GCG* may contribute to the development of obesity. For diabetics, the top gene predicted by GenePANDA is *POMC* that encodes a neuropeptide called proopiomelanocortin. In brain, proopiomelanocortin (POMC) neurons are vital within the hypothalamic arcuate nucleus that control appetite and feeding^40^. A recent study showed that dysfunction of POMC neurons upon high-fat consumption is a major pathogenic mechanism involved in the development of obesity and type 2 diabetes mellitus^41^. *POLD1* is the top predicted gene for breast cancer by GenePANDA. It encodes p125, the catalytic subunit of human DNA polymerase delta^42^,^43^. Human p125 modulates cell cycle progression and therefore promotes the cancer proliferation^44^. In addition, a recent study observed increased methylation of *POLD1* promoter and increased expression of p125 in breast cancer cell lines and tissues^45^, suggestive of a link of *POLD1* to breast cancer development.

**Figure 3:**
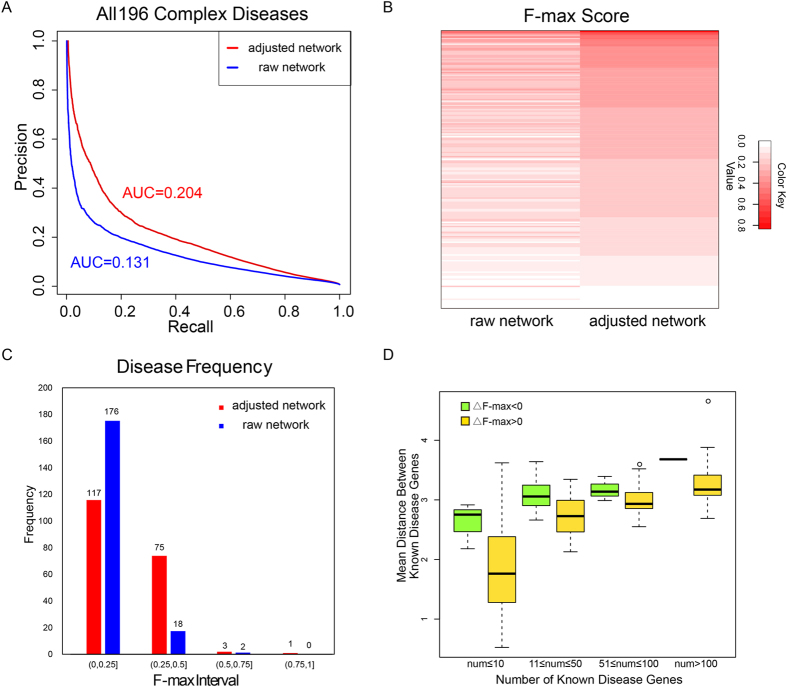
Prediction performance of GenePANDA on 196 diseases. Prediction scores for each disease based on adjusted network distance or raw network distances are combined together to plot the Precision-Recall curves, with the corresponding values of the area under curve (AUC) shown above each curve (**A**). The F-max scores for each of the 196 diseases using adjusted network distance (score in descending order, right) or raw network distance (corresponding to former, left) shown in heat map (**B**). The histogram of diseases at different intervals of F-max scores is shown in (**C**). (**D**) shows the boxplots of the mean raw network distance between disease genes for those diseases with better or worse F-max scores after the use of adjusted network distance. Diseases are grouped according to the number of annotated genes.

Having evaluated the performance of GenePANDA in the above three diseases, we next conduct leave-one-out cross validation for predicting disease genes for each of 196 complex diseases. In order to make the prediction scores comparable across diseases, for each disease we apply the score conversion procedure to convert the disease-specific gene weights into probability scores. To gain an overall understanding of GenePANDA’s performance on prioritizing disease genes, we combine all gene-disease predictions (19,038 × 196 ~ 3.7 million prediction scores) to plot the precision-recall curve. The AUC of the precision-recall curve is 0.204. In contrast, replacing the adjust network distance with the raw network distance would lead to an AUC of 0.131 (Fig. 3A). Thus, the use of adjusted network distance significantly improves the performance of GenePANDA for prioritizing disease genes (an improvement of over nearly 56% over the use of raw network distance).

We also investigate the performance of GenePANDA on individual diseases by calculating the respective F-max scores. The F-max score can be interpreted as a weighted average of the precision and recall, with a higher F-max score indicating a superior overall performance. For example, the disease with the best F-max score (0.833) by GenePANDA using adjusted network distance is iron overload. We also compare the F-max scores produced by using adjusted network distance with that by using raw network distance, and find that for 184 out of 196 diseases, the use of adjusted network distance results in a higher F-max score (Fig. 3B). The disease with the most improvement in F-max score is osteosarcoma, with an improvement of over 23-fold (from 0.014 to 0.333) after distance adjustment. Specifically, about 41% (79 out of 196) of diseases have a F-max-score greater than 0.25 using the adjusted network distance, in contrast to only about 10% (20 out of 196) using the raw network distance (Fig. 3C). Though the use of adjusted network distance has improved the prediction performance for most diseases, there are still a few whose F-max scores become worse with the use of adjusted network distance. Considering that in general diseases with higher number of disease genes tend to have better F-max scores, we divide 196 diseases into four categories based on the number of their disease genes (< = 10, 11 ~ 50, 51 ~ 100, and >100 disease genes). In all four categories, we find those diseases with worse F-max scores after the use of adjusted network distance have significantly higher mean raw network distance between their disease genes than those with improved F-max scores (Fig. 3D). In the other words, for those diseases with worse F-max scores, the disease genes tend to be more interspersed in the network. Thus, the adjusted network distance approach may not be effective for those diseases with scattered topology in the network.

### GenePANDA shows superior performance for gene prioritization over five existing methods

Over the years, many methods have been developed for prioritizing disease genes. These methods can be generally classified into three major categories: data mining, similarity profiling, and network analysis-based methods. Data mining-based methods typically rely on the mining of literature data, while similarity profiling and network analysis-based methods both explore functional genomics data. Here, we compare GenePANDA with three network analysis-based methods—Maxlink^36^, Genefriends^25^ and Pinta^23^ and two similarity profiling-based methods—Endeavour^21^ and Candid^34^.

**Figure 4:**
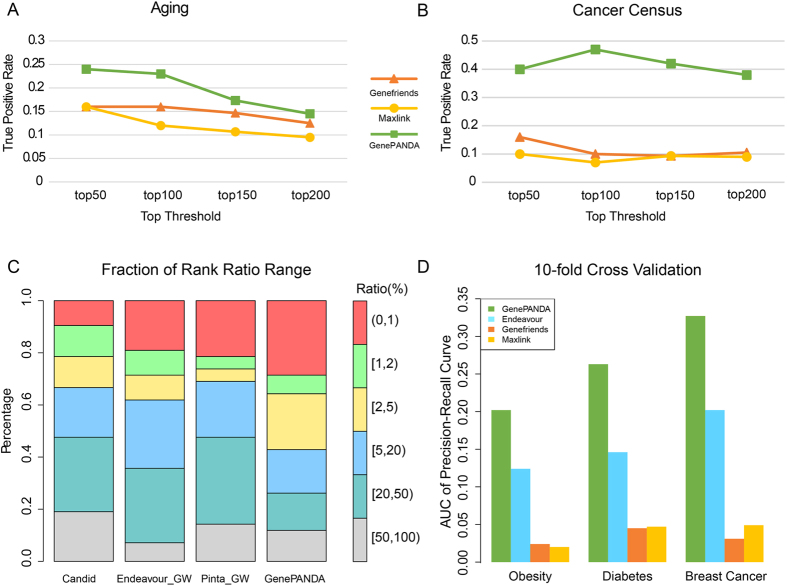
The comparison of GenePANDA with five published methods. (**A**) and (**B**) show the true positive rate at different rank threshold of the predictions made by GenePANDA, Maxlink, and Genefriends for predicting aging and cancer related genes, respectively. (**C**) shows the rank ratios distribution of the 42 novel disease-gene associations predicted by GenePANDA, Candid, Endeavour-GW, and Pinta-GW. The corresponding numbers for Candid, Endeavour-GW, and Pinta-GW are obtained from Bornigen *et al*.^33^. (**D**) shows the summarized result of the AUC of precision-recall curves on obesity, diabetes and breast cancer after 10-fold cross validation using GenePANDA, Endeavour, Genefriends and Maxlink.

We first compare GenePANDA with Maxlink and Genefriends because these two methods have available webservers, and their respective papers also provided lists of disease genes (cancer and aging, respectively) for benchmarking. We then carry out leave-one-out cross validation with input of two lists of genes using GenePANDA, Maxlink and Genefriends, separately. To evaluate prediction performance, we compute the true-positive rates (TPR) at the top 50, 100, 150 and 200 predictions for each method. In both cancer and aging, GenePANDA achieves superior performance over Maxlink and Genefriends at each of the rank thresholds (Fig. 4A,B). For example, it achieves a TPR of 24% and 23% for aging at top 50 and 100 predictions, respectively, in contrast to 16% and 16% for Genefriends and 16% and 12% for Maxlink, respectively (Fig. 4A). For cancer genes, GenePANDA achieves TPR of 40% and 47% at top 50 and top 100 predictions, respectively, much higher than that by Genefriends (16% and 10%) and Maxlink (10% and 7%) (Fig. 4B).

Recently, Bornigen *et al*. have compiled 42 novel disease-gene associations from literatures, and used these associations as unbiased validation for evaluating the performance of a number of disease gene prioritization methods, including Candid, Endeavour-GW, and Pinta-GW^33^. Bornigen *et al*. also provided the respective lists of input disease genes corresponding to each of the 42 disease-gene associations. Here, we run GenePANDA using each of the 42 lists of input disease genes, and carry out genome-wide predictions. Then, we follow Bornigen *et al*. to rank all genes according to their prediction scores for a given disease (from higher to lower), and compute the rank ratio of the 42 novel disease genes (the rank of the novel disease gene divided by the total number of predicted genes, with lower rank ratio indicating better performance). We also obtain the rank ratios of the 42 novel disease genes by Candid, Endeavour-GW, and Pinta-GW from Bornigen *et al*. Among the four methods being compared, GenePANDA achieves the highest response rate at all rank ratio thresholds (Fig. 4C) (for details, refer to Supplementary Table S2). The median of the rank ratios for the 42 novel disease-gene associations by GenePANDA is 17.8%, the best among the four methods (Candid (27.3%), Endeavour-GW (21.5%) and Pinta-GW (23.5%)).

In addition, we apply Endeavour, Genefriends and Maxlink using the default settings to conduct 10-fold cross validation on obesity, diabetes and breast cancer (Candid and Pinta are not tested because Candid requires keywords as input, while Pinta requires expression data). The comparison of the AUC of the precision-recall curves based on different methods shows that GenePANDA achieves the best performance on all these three diseases (Fig. 4D). For example, for obesity the AUC produced by GenePANDA is 0.202, significantly higher than that by Endeavour (0.124), Genefriends (0.024), and Maxlink (0.020).

In conclusion, based on all above benchmarks GenePANDA achieves superior performance for prioritizing disease genes over the five methods being compared.

### Application of GenePANDA for prioritizing SNPs identified by GWAS

A typical GWAS may produce hundreds or more of SNPs that are significantly associated with the disease of interest. However, only a small proportion of the SNPs identified by GWAS are functional polymorphisms that contribute to disease phenotypes^46^. Prioritizing SNPs of functional importance is therefore of significant value for post-GWAS investigation. Typically, researchers will focus on those non-synonymous SNPs whose host genes are known disease genes. Since current knowledge about disease genes is often not complete, this will miss the opportunity to uncover novel functionally important SNPs. Below we show that by using GenePANDA to predict candidate disease genes, we can further identify likely functionally important SNPs that would be otherwise missed in post-GWAS studies.

The GWAS databases corresponding to Crohn’s disease, obesity and rheumatoid arthritis have collected 29, 21 and 103 disease-associated non-sysnoymous SNPs (daSNPs) from a number of GWAS studies, respectively. Among these daSNPs, the host genes of 13, 18 and 63 are known disease genes. By predicting candidate disease genes for these three disease using GenePANDA, we further identify 2, 1 and 1 likely novel functionally important SNPs for these SNPs by requiring their host genes be among the top 500 predictions. The two SNPs for Crohn’s disease are rs12720356 and rs4077515 whose host genes (*TYK2* and *CARD9*) rank 14^th^ and 354^th^ by GenePANDA. *TYK2* encodes a member of Janus kinases protein families that are intracellular nonreceptor tyrosine protein kinases, and play key roles in regulating immune cell function. It has been shown that therapies directed against Janus kinases are promising alternative approaches for Crohn’s disease in recent years^47^,^48^. As for *CARD9*, a recent study found a novel rare SNV (rs200735402) in *CARD9* that have a protective effect for Crohn’s disease in Korean population^49^, indicating a link of *CARD9* to Crohn’s disease. The prioritized SNP for obesity is rs11676272 that locates on *ADCY3. ADCY3* ranks 461^st^ in obesity prediction list. Adenylate cyclase 3 (ADCY3) is the third member of adenylyl cyclase family and catalyses the synthesis of cAMP from ATP. Epigenetic studies have indicated that increased DNA methylation levels in the *ADCY3* gene are involved in the pathogenesis of obesity^50^,^51^,^52^. The prioritized SNP for rheumatoid arthritis is rs2071888 that locates on *TAPBP. TAPBP* ranks 325^th^ among the predicted rheumatoid arthritis genes by GenePANDA. It encodes Tapasin, an MHC class I molecule, that was found to excessively express in the bone marrow cells of rheumatoid arthritis patients, and was considered to play key roles in the abnormal regulatory networks in immune response to rheumatoid arthritis^53^. As such, using GenePANDA to predict candidate diseases, we can help to identify likely functional importance SNPs that would be otherwise ignored in post-GWAS studies.

## Discussion

GenePANDA is a novel network analysis-based method for prioritizing disease genes. It differentiates from other network analysis-based methods in two major aspects—the use of adjusted network distance, and the way for calculating disease-specific gene weights. We validate the performance of GenePANDA through literature reviews, and also show GenePANDA’s superiority over five existing methods using two benchmarks. Finally, GenePANDA is shown to be of use for prioritizing SNPs identified by GWAS. We have constructed an online GenePANDA webserver which provides not only the lists of candidate disease genes for 196 complex diseases for downloading, but also a web interface for users to provide user-defined disease genes and run GenePANDA to predict candidate disease genes.

GenePANDA relies on a functional interaction network to predict candidate disease genes. The quality of the functional interaction network therefore may potentially affect the performance of GenePANDA. In this study, the STRING network used by GenePANDA is version 9.1. The latest version is 10.0, which shows significant difference than version 9.1: it includes over 8.5 million interactions, in contrast to about 4.8 million interactions in version 9.1; the common interactions only account for 37.8% and 21.4% of the interactions in version 9.1 and 10.0, respectively, and have significantly different scores in the two versions (p-value < 2.2e-16, KS test). Despite the significant difference of individual interactions in the two versions, GenePANDA produces similar results using the two versions based on 10-fold cross validation on 196 complex diseases: the AUC of the precision-recall curve is 0.170 and 0.114 based on adjusted and raw network distances using version 10.0, respectively, while the corresponding AUC using version 9.1 is 0.189 and 0.110, respectively (Supplementary Fig. S1). The fact that GenePANDA is robust against significant changes on individual interactions implies that the underlying network topology for a given disease remains similar even though there may be significant difference in individual interactions, an important merit for network-based methods such as GenePANDA. Besides the STRING network, there are also a number of large-scale functional interaction networks that differ from STRING in their knowledge source and scoring strategy, such as Funcoup, PIPs^54^, Genes2FANs^55^, GeneMania^56^ and HEFalMp^57^, etc. The integration of different functional interaction network, or the combination of predicted candidate disease genes using different networks may help further improve the performance of GenePANDA. On the other hand, the performance of GenePANDA is largely dependent on the network topology of different diseases, with it generally being more effective for those diseases with relatively compact network topology. The disease gene annotations used in this study are from Genetic Association Database (GAD), which has stopped to be updated in 2014. The inclusion of more disease gene annotations from other sources, such as DisGeNET^58^, Phenopedia^59^ and BeFree^60^, may change some disease’s network topology, and improve GenePANDA’s prediction performance.

In this study, we have shown GenePANDA’s predictions can be helpful for prioritizing SNPs identified by GWAS. The predicted candidate disease genes can also be applied to prioritize disease genes for resequencing or designing knock-in/out experiments. Or, they can be used for designing custom gene panel for genetic testing of complex diseases, and for assisting in the identification of disease variants produced by whole exome sequencing on patients with rare diseases. In addition, GenePANDA is not limited to predict only disease genes. Given a list of genes sharing a common characteristic, such as the same phenotype or the same function, GenePANDA can be readily applied to conduct genome-wide survey for more genes that potentially have the same characteristic. What’s more, GenePANDA can be considered as a general framework, and be used for predicting other candidate functional elements, such as miRNAs or lncRNAs, that have disease phenotypes, as long as a functional interaction network can be constructed for miRNAs or lncRNAs as well.

## Additional Information

**How to cite this article:** Yin, T. *et al*. GenePANDA—a novel network-based gene prioritizing tool for complex diseases. *Sci. Rep.* **7**, 43258; doi: 10.1038/srep43258 (2017).

**Publisher's note:** Springer Nature remains neutral with regard to jurisdictional claims in published maps and institutional affiliations.

## Supplementary Material

Supplementary Information
