# Supplementary material for: GenePANDA—a novel network-based gene prioritizing tool for complex diseases
Source: Sci Rep. 2017 Mar 2;7:43258. doi: 10.1038/srep43258 (PMC5333103; doi:10.1038/srep43258)
Supplement: Supplementary Information [file srep43258-s1.pdf]

# GenePANDA—a novel network-based gene prioritizing tool for complex diseases

Tianshu Yin[1,2], Shu Chen[1,3], Xiaohui Wu[1,3], Weidong Tian[1, 2*]

Authors' affiliations:

[1]State Key Laboratory of Genetic Engineering and Collaborative Innovation Center for Genetics and Development, School of Life Sciences, Fudan University, Shanghai 200436, P.R. China

[2]Department of Biostatistics and Computational Biology, School of Life Sciences, Fudan University, Shanghai 200436, P.R. China

[3]National Center for International Research of Development and Disease, Institute of Developmental Biology and Molecular Medicine, Fudan University, Shanghai 200433, P. R. China

*Corresponding Authors

Email:

Tianshu Yin: tsyin10@fudan.edu.cn

Shu Chen: chenshu@fudan.edu.cn

Xiaohui Wu: xiaohui_wu@fudan.edu.cn

Weidong Tian: weidong.tian@fudan.edu.cn (Corresponding author)

**Supplementary Table S1**

| Top 10 predicted genes whose association with obesity have been reported recently | | |
|---|---|---|
| **Gene** | **Rank** | **Literature Experimental Evidence Pubmed ID** |
| GCG | 1 | 26186884 |
| GAL | 2 | 26172564 |
| SST | 3 | 25956467 |
| KNG1 | 4 | NA |
| CPB2 | 5 | NA |
| GCK | 6 | 26132169 |
| LEPR | 7 | 26365669 |
| IAPP | 8 | 26398307 |
| PPY | 9 | 26612764 |

| | | |
|---|---|---|
| PNPLA2 | 10 | 26500201 |
| Top 10 predicted genes whose association with diabetes have been reported recently | | |
| **Gene** | **Rank** | **Literature Experimental Evidence Pubmed ID** |
| PRO2044 | 1 | NA |
| POMC | 2 | 26930171 |
| NOS3 | 3 | 25477161 |
| PRL | 4 | 26299069 |
| TTR | 5 | 25781688 |
| SST | 6 | 26589890 |
| TGFBI | 7 | 24728038 |
| SCARB2 | 8 | NA |
| AGT | 9 | 26380312 |
| REN | 10 | 26569322 |
| Top 10 predicted genes whose association with breast cancer have been reported recently | | |
| **Gene** | **Rank** | **Literature Experimental Evidence Pubmed ID** |
| POLD1 | 1 | 27022290 |
| BIRC5 | 2 | 26372358 |
| FOS | 3 | 26536102 |
| RFC5 | 4 | NA |
| ATR | 5 | 26319584 |
| CHK2 | 6 | 26025911 |
| RFC4 | 7 | NA |
| RFC3 | 8 | NA |
| SP1 | 9 | 26645832 |
| GADD45B | 10 | 25840970 |

**Supplementary Table S2**

| Rank Ratio of 42 Novel Associations by 4 Methods | | | | |
|---|---|---|---|---|
| | Candid | Endeavour-GW | Pinta-GW | GenePANDA |
| ACAD9 | 33.45 | 1.53 | 31.53 | 8.14 |
| ATF7IP | 1.38 | 49.88 | 58.21 | 35.48 |
| BCL3 | 60.27 | 4.74 | 10.76 | 9.89 |
| BRCA2 | 1.37 | 1.25 | 0.29 | 8.50 |
| C20orf54 | 67.5 | 95.54 | 95.04 | 0.17 |
| CD320 | 80.54 | 46.95 | 26.41 | 0.05 |
| CHST14 | 61.64 | 7.57 | 25.8 | 0.26 |
| CRHR1 | 15.04 | 13.35 | 12.94 | 11.87 |
| CSF1R | 2.93 | 1.67 | 2.69 | 2.65 |

| | | | | |
|---|---|---|---|---|
| DAB2IP | 14.2 | 26.4 | 21.03 | 26.80 |
| DISP1 | 2.26 | 32.85 | 93.23 | 7.23 |
| DMRT1 | 41.83 | 79.04 | 29.94 | 8.76 |
| EZH2 | 4.66 | 34.43 | 23.41 | 4.69 |
| FUT2 | 47.67 | 42.43 | 19.74 | 14.30 |
| G6PC3 | 47.72 | 18.72 | 51.22 | 71.53 |
| GABRR1 | 17.43 | 15.97 | 11.75 | 12.66 |
| GLIS3 | 7.92 | 2.25 | 0.76 | 29.54 |
| HCCS | 25.85 | 2.75 | 10.78 | 89.21 |
| HTR7 | 17.7 | 0.56 | 1.73 | 5.18 |
| IFNG | 47.19 | 1.21 | 0.21 | 0.16 |
| IL10 | 13.22 | 26.26 | 0.18 | 0.24 |
| LPP | 74.55 | 6.93 | 17.62 | 2.87 |
| MECOM | 11.6 | 15.89 | 30.24 | 11.71 |
| MMEL1 | 25.2 | 39.37 | 18.32 | 6.49 |
| PLCE1 | 10.45 | 13.03 | 42.6 | 13.04 |
| PQBP1 | 22.51 | 16.74 | 34.79 | 83.61 |
| RANBP1 | 7.32 | 46.07 | 48.2 | 91.66 |
| RUNX2 | 1.79 | 3.07 | 0.18 | 0.11 |
| SDCCAG8 | 78.11 | 5.23 | 0.85 | 0.12 |
| SH2B1 | 3.53 | 10.82 | 12.64 | 12.02 |
| SOX17 | 74.67 | 15.08 | 1.11 | 1.82 |
| SPIB | 5.56 | 10.28 | 30.44 | 24.71 |
| STOM | 26.54 | 6.52 | 22.72 | 22.06 |
| TBX2 | 31.34 | 1.87 | 1.34 | 1.87 |
| TNFRSF19 | 5.74 | 36.09 | 21.24 | 10.81 |
| TP63 | 7.04 | 1.35 | 11.67 | 5.50 |
| TRAF3IP2 | 2.05 | 39.04 | 23.1 | 9.69 |
| TRAF6 | 0.13 | 16.54 | 8.11 | 1.81 |
| UBE2E2 | 51.18 | 17.38 | 61.89 | 69.03 |
| UBE2L3 | 49.17 | 71.21 | 6.34 | 33.65 |
| UTRN | 18.51 | 0.46 | 2.99 | 1.08 |
| WDR62 | 29.98 | 23.35 | 63.97 | 0.01 |

**Supplementary Figure S1**

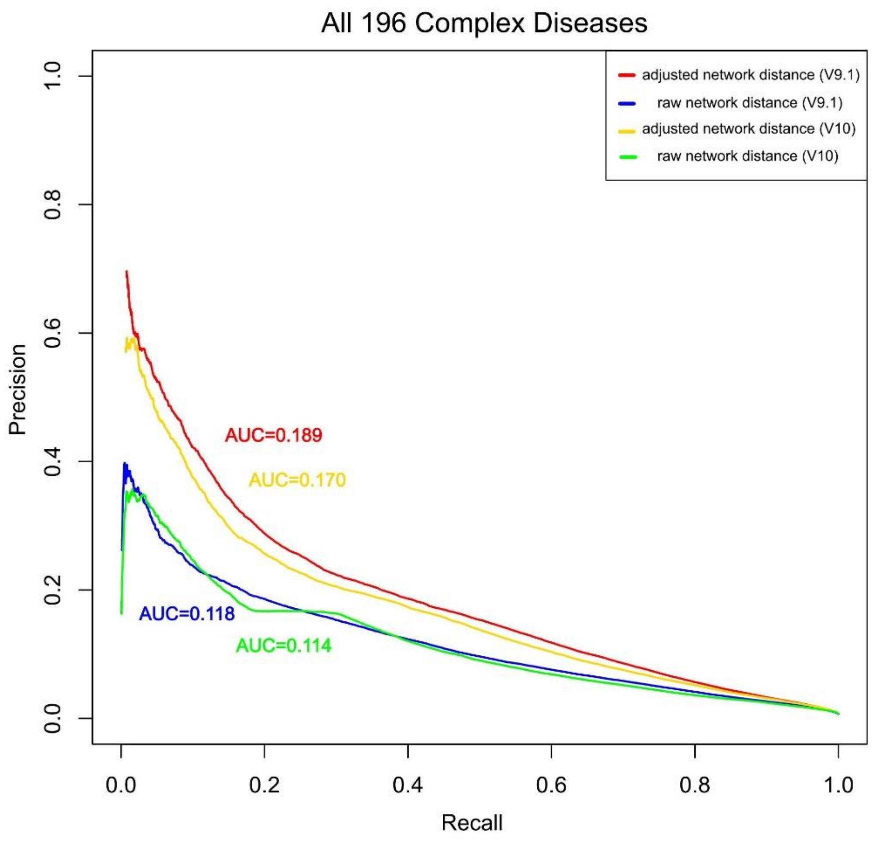


**Supplementary Figure S1:** Prediction performance of GenePANDA on 196 diseases on adjusted and raw network distance based on STRING v9.1 and STRING v10.
